# Bilateral symmetry of the subtalar joint facets and the relationship between the morphology and osteoarthritic changes

**DOI:** 10.1002/ca.23525

**Published:** 2019-12-06

**Authors:** Roeland P. Kleipool, Gwendolyn Vuurberg, Sjoerd A.S. Stufkens, Alie E. van der Merwe, Roelof‐Jan Oostra

**Affiliations:** ^1^ Department of Medical Biology, Amsterdam UMC, Academic Medical Center University of Amsterdam, Amsterdam Movement Sciences Amsterdam The Netherlands; ^2^ Academic Center for Evidence‐Based Sports Medicine (ACES) Amsterdam The Netherlands; ^3^ Department of Orthopaedic Surgery, Amsterdam UMC, Academic Medical Center University of Amsterdam, Amsterdam Movement Sciences Amsterdam The Netherlands; ^4^ Amsterdam Collaboration on Health & Safety in Sports (ACHSS), Amsterdam UMC, IOC Research Center Amsterdam The Netherlands

**Keywords:** ankle anatomy, subtalar joint, osteoarthritis, bone symmetry

## Abstract

There is a paucity in the literature regarding bilateral symmetry between the facets of the subtalar joint. Often surgeons use the contralateral side as a reference when dealing with a fracture or other joint pathology. Moreover, the presence of osteoarthritic (OA) changes in the subtalar joint is suggested to have a relation with its morphology. In this study, we addressed both these issues. Forty pairs of cadaveric tali and calcanei were analyzed by dissection and measurement. Twenty pairs of asymptomatic calcanei were morphologically analyzed by computer tomography imaging. In the cadaveric feet, the length and width of the facets, the number and interfacet connections, the intersection angle, and the presence of OA changes were registered. In the healthy feet, the orientation and curvature of the posterior facet were analyzed based on cylinder fittings. Bilateral symmetry was tested with paired Student's *t* tests. Significant associations between morphometric parameters and the presence of OA changes were tested with generalized estimating equation logistic regression models. The morphometric data demonstrated a high degree of bilateral symmetry. The types of tali and calcanei between left and right differed in about one‐fifth of the individuals. No significant interactions were found between morphological parameters and the presence of OA changes. Only age had a significant association. There was a high degree of symmetry in the subtalar joints facets. No significant associations were found between OA changes and morphological features, whereas other studies did. Further research is needed to explore this relationship in further detail. Clin. Anat., 33:997–1006, 2020. © 2019 Wiley Periodicals, Inc.

## INTRODUCTION

In most classical anatomy textbooks, the talocalcaneal joint has three distinct articulating facets. However, it has been well documented that interindividual variation exists in the number, the distance, between angle and connections, and the size of the articulating joint facets (Bruckner, 1987; Forriol Campos and Gomez Pellico, 1989; Drayer‐Verhagen, 1993; Barbaix et al., 2000; Ragab et al., 2003; Madhavi et al., 2008; Jung et al., 2015; Agarwal et al., 2016). Despite the known variation only two studies analyzed the degree of bilateral symmetry of the facet joints: one describing the pattern of calcaneal facets (Ragab et al., 2003), and another one describing the surface area and three‐dimensional (3D) orientation of the calcaneal facets (Stephan et al., 2014). In general, literature describing the bilateral symmetry of the hindfoot is scarce (Shultz and Nguyen, 2007; Islam et al., 2014; Tümer et al., 2018). This is remarkable since many surgeons use the contralateral noninjured side as reference for anatomical reduction in case of a fractured bone (Pierre et al., 2010; Dobbe et al., 2011; Young et al., 2013; Ten Berg et al., 2016). Even less literature is available describing the symmetry of the articulating facets. This is possibly even more important, since the geometry of the articular surfaces primarily determines the kinematics (Kleipool and Blankevoort, 2010). The first objective of the present study was therefore to evaluate and describe the degree of bilateral symmetry present in the pattern and size of articulation facets involved in the talocalcaneal joint.

Although often overlooked in clinical practice, the talocalcaneal joint has also been hypothesized to play a role in multiple ankle pathologies, such as planovalgus and cavovarus deformity, coalitions, chronic ankle instability, and so on (Aynardi et al., 2015). The morphology of the joint forms a major component in the joint's stability (Stormont et al., 1985). Bruckner (1987) was the first to hypothesize that a “three‐facet” configuration contributes to a greater talocalcaneal stability, as the talus is supported by an “osseous tripod.” Drayer‐Verhagen (1993) and Madhavi et al. (2008) confirmed this by showing less osteoarthritic (OA) changes associated with the “three‐facet” configuration. Not only the number of facets, but also the angle of the anterior and middle facets in a sagittal plane (intersection angle) has been related to OA changes (Drayer‐Verhagen, 1993; Agarwal et al., 2016). The aforementioned studies only focused on the calcaneus and did not include the talus. A second objective of the present study was therefore to further explore the hypothesis of a relationship between the morphology of the subtalar joint facets and its stability and to evaluate the morphology as a predictive factor for developing OA.

## MATERIALS AND METHODS

For the present study two data sets were used. The first data set was acquired for the present study, and the second comes from a previous study (Kleipool et al., 2019).

### Bilateral Symmetry

A convenience sample of a total of 40 pairs (21 male and 19 female) of cadaveric ankles of known age and sex were collected for assessment after a dissection course for first years bachelor medical students (Table 1; mean age at time of death = 79 years ±13 SD, range 48–104 years). The material originated from the body‐donation program of the Amsterdam UMC, location AMC in accordance with Dutch law article 21 of the Burial and Cremation Act (BWBR0005009) and the regulations of the medical ethical committee of the institution. Although ancestry is not recorded for donations, a survey among the donors in The Netherlands showed that up to 98% was of native Dutch origin (Bolt et al., 2010). A tentative assumption can therefore be made that the sample investigated represents a Western‐European population.

**Table 1:** Age distribution (in years) at time of death/at time of scan of all specimen/individuals included in the study, divided into 10 years age intervals

Age	20–29	30–39	40–49	50–59	60–69	70–79	80–89	90–99	100–109
Cadaveric specimen			1	1	7	11	10	9	1
Living individuals	6	9	2	3					

The hindfoot was isolated from the body by a transverse section through the leg approximately 10 cm proximal from the medial malleolus. Next, the subtalar joint was opened at the lateral side and unfolded to the medial side. Hereto, a deep incision was made from the dorsomedial side of the foot at the level of the talonavicular joint to the inferior tip of the lateral malleolus and from here through the Achilles tendon. The lateral tendons, ligaments, and joint capsules were cut, including the connections in the tarsal sinus and canal. Subsequently, the subtalar joint could be opened with relative ease.

**Figure 1:**
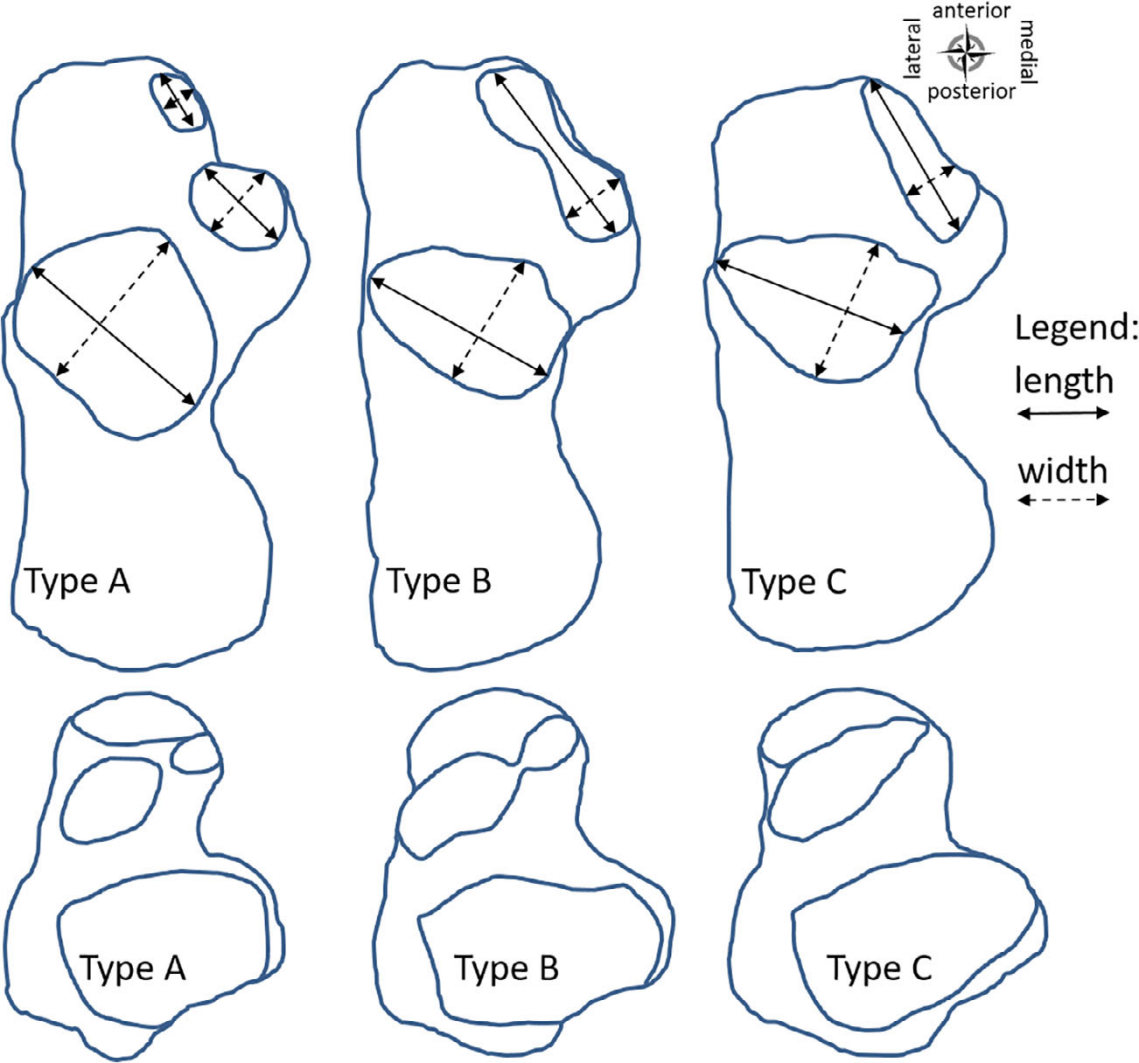
Contour drawings of the different types of calcanei and tali based on the number and connections of the facets. Type A: All three facets are separated. Type B: The posterior facet is separated from the middle, but the middle is connected to the anterior facet by a small bridge. Type C: The posterior facet is separated, but the anterior and middle facets form one facet. Calcaneus drawn from a superior view. Talus drawn from an inferior view. The length and the width of the facets were measured as illustrated with the bidirectional arrows (length: solid line; width: dashed line). [Color figure can be viewed at wileyonlinelibrary.com]

Many classifications for the different patterns of the talar and calcaneal facets are found in the literature. In the present study, the classification of Jung et al. (2015) was used (Fig. 1).

- Type A: All three facets are separated.
- Type B: The posterior facet is separated from the middle, but the middle is connected to the anterior facet by a small bridge. At this constriction between the middle and anterior facets is a slope discontinuity or an obvious angle.
- Type C: The posterior facet is separated, but the anterior and middle form one facet. The single facet has a smooth, rounded surface with a slight convexity at the talar head and a slight concavity in the calcaneus.

Two other types, a variant where the anterior facet is absent and one where all facets are connected, were not found in this study sample and therefore not included (Bunning and Barnett, 1965; Bruckner, 1987; Drayer‐Verhagen, 1993). The classification system described above facilitates comparison of the results of the present study with the results of Drayer‐Verhagen (1993) and Madhavi et al. (2008).

Two independent observers assessed the pattern of the calcaneal and talar facets. If the two observers disagreed the observation of Observer 1 was included in further analyses. This was arbitrarily chosen. Also, both observers performed the following morphometric measurements of the calcaneus with a sliding vernier caliper, accurate to 0.1 mm. The length of the posterior, middle, and anterior facets was measured in an anterolateral to posteromedial direction along the long axis of the facets. The width was measured perpendicular to this long axis (Fig. 1). In case of a Type B or C the total length of the whole (fused) facet was measured and the width perpendicular to this line at the widest location. The minimal distance between the posterior and middle (in Type A) or fused middle and anterior facets (in Type B or C) was measured in all calcanei, and between the anterior and middle only in Type A calcanei.

Next, the osseous calcanei were isolated by maceration in hot water with common household dish soap for several days. The remaining soft tissue, including the cartilage, was scrubbed off from the calcanei.

The intersection angle between the anterior and middle facets was determined by placing the macerated calcaneus on its medial side on a piece of paper (Drayer‐Verhagen, 1993; Jung et al., 2015; Agarwal et al., 2016). The medial border of the sustentaculum tali facets contacted the paper, and the planes of the facets were manually orientated perpendicular to the surface of the paper. The contour of the facets was traced with a pencil. Subsequently, the image was digitized by photoscanning the paper and the intersection angle was accordingly measured on the digital image using the angle measurement tool of the imaging software ImageJ (U.S. National Institutes of Health, Bethesda, MD).

**Figure 2:**
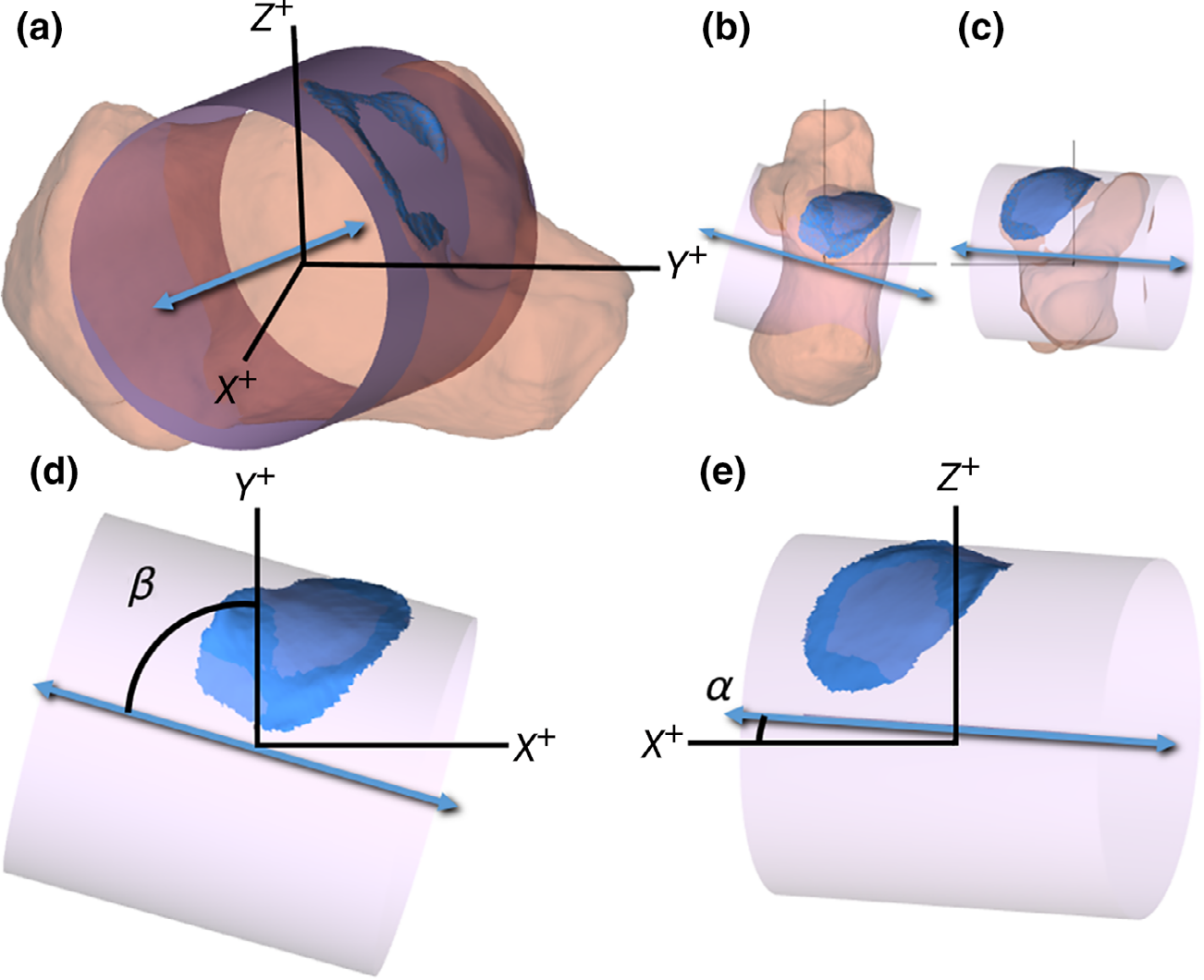
Graphical representation of a surface model of a right calcaneus (brown) and the posterior calcaneal facet of the talocalcaneal joint (in blue). A cylinder was fitted to the posterior facet (pink). The orientation of the cylinder's axis (blue bidirectional arrow) was defined by the inclination angle (alpha) and the deviation angle (beta). **a**, Anterolateral view; **b**, superior view; **c**, anterior view; **d**, magnification of the same superior view as in **b** without the calcaneus; and **e**, magnification of the same anterior view as in **c** without the calcaneus. [Color figure can be viewed at wileyonlinelibrary.com]

As mentioned in the beginning of this section, data from a previous study on living individuals were also included (Kleipool et al., 2019) to account for the 3D morphology of the posterior facet compared to measurements taken with a sliding caliper in the cadaveric specimens. In the previous study (approved by the Amsterdam UMC, location AMC Internal Review Board, registration number NL60684.018.17), 20 (10 male and 10 female) left and right healthy and nonsymptomatic calcanei were segmented from computer tomography (CT) scans with custom made software (Dobbe et al., 2011; average age at time of scan = 35.9 years ±11.1 SD, range 23–59 years, Table 1). The surface of the posterior facet was identified and isolated from the acquired surface model of the whole calcaneus. All left calcanei and facets were mirrored to right calcanei and facets for a corresponding orientation. With custom‐made routines in Matlab (MATLAB Release 2016a, The MathWorks, Inc., Natick, MA) a cylinder was fitted to the posterior facet using a nonlinear least‐squares optimization process with the axis in a predominantly transverse orientation. The orientation of the cylinder's axis was expressed by two angles in reference to a coordinate system based on the geometric principal axes of the subject's calcaneus (Fig. 2). The inclination angle was defined as the angle between the cylinder's axis and the *XY*‐plane. The deviation angle was defined as the angle between the projection of the cylinder's axis on the *XY*‐plane and the *Y*‐axis, or the sagittal plane. A negative inclination angle corresponds to an inferiorly or plantarly orientated axis. A negative deviation angle corresponds to a medially orientated axis (Fig. 2). Also the diameter of the cylinder fit was determined as a measure for the curvature of the posterior facet. For further details, see Kleipool et al. (2019).

### Osteoarthritic Changes

After opening the subtalar joint of the cadaveric specimen by dissection of the intact specimen, prior to removal of the soft tissue by maceration, the presence of OA changes was scored on both the talar and the calcaneal facets by one observer (orthopedic researcher). This was performed with the unaided eye. OA changes were scored as absent/present regardless of the severity of the defects. The location of defects was recorded as posterior, middle, or anterior (also in calcanei B and C) regardless of the facet configuration.

### Statistics

The intraclass correlation coefficient (ICC) was calculated as a measure of interobserver reliability. The ICC was interpreted as poor (≤0.40), moderate (0.40–0.75), substantial (0.75–0.90), or excellent reliability (>0.90) (Terwee et al., 2007).

The Stuart–Maxwell tests of marginal homogeneity were used to test for significant left–right differences for the type of bone. Morphometric differences between the left‐ and right‐sided facets were assessed using a paired Student's *t* test. An analysis of variance (ANOVA) was performed to explore differences between the intersection angle of the three types. A Bonferroni post hoc analysis was performed if the ANOVA indicated a significant difference.

Generalized estimating equation (GEE) logistic regression models were made to identify the factors associated with the presence of OA changes on the calcaneus. A binomial distribution for OA changes was used with subject identifier as repeated effect and an unstructured correlation matrix. In the univariable model, significant interaction was investigated for age (per 5 years), intersection angle, type of calcaneus or talus, matching pattern between talus and calcaneus (on a binary scale; corresponding or noncorresponding type), and dimensions (length and width) of the facets.

Statistical significance was accepted at *P* < 0.05. All analyses were performed using SPSS statistical software (IBM SPSS Statistics for Windows, Version 24.0, Released 2015, IBM Corp., Armonk, NY).

## RESULTS

### Left–Right Analyses

#### *Facets pattern*

The cadaveric calcanei and tali were classified as Types A, B, or C based on the calcaneal and talar facets pattern of connection and number (Fig. 1). Most of the specimens had the same type between the left and right sides. In total, 77.5% of the calcanei and 80% of the tali had bilateral symmetry in their type (Table 2). The remaining pairs of calcanei (22.5%) and tali (20%) had varying combinations of types between the left and right sides (Table 2). The most frequently observed asymmetry was a combination of Types A and B within individuals. The differences found between the left and right sides in type, for either the calcanei or tali, were, however, statistically nonsignificant.

**Table 2:** Cross tabulation of types of cadaveric calcanei and tali (Types A, B, or C) based on the patterns of the calcaneal and talar facets for the left and right sides

	Left	Right						Total left	
	Type A		Type B		Type C				
Calcaneus	Type A	**22.5%**	**(9)**	5.0%	(2)	–	–	*27.5%*	*(11)*
	Type B	10.0%	(4)	**47.5%**	**(19)**	5.0%	(2)	*62.5%*	*(25)*
	Type C	–	–	–	–	**10.0%**	**(4)**	*10.0%*	*(4)*
Total right		*32.5%*	*(13)*	*52.5%*	*(21)*	*15.0%*	*(6)*	*100%*	*(40)*
Talus	Type A	**57.5%**	**(23)**	7.5%	(3)	–	–	*65.0%*	*(26)*
	Type B	7.5%	(3)	**5.0%**	**(2)**	5.0%	(2)	*17.5%*	*(7)*
	Type C	2.5%	(1)	–	–	**15.0%**	**(6)**	*17.5%*	*(7)*
Total right		*67.5%*	*(27)*	*12.5%*	*(5)*	*20.0%*	*(8)*	*100%*	*(40)*

The diagonal with bold numbers represent the corresponding classifications between the left and right side. Off diagonal numbers represent the noncorresponding classifications. Numbers presented are the percentages of the total, and the numbers in parentheses represent the absolute number of specimen.

#### *Metric data calcaneal facets*

Both the metric assessment of the calcaneal facets as well as the curvature and orientation of the posterior facet by means of a cylinder fit to the facet's surface were assessed in the analysis of left–right symmetry. All parameters, for both data sets, showed a strong degree of symmetry between the left and right sides (Table 3). No significant differences were found between the left and right sides for all morphometrics of the calcaneal facets, except for the width of the posterior facet (*P* = 0.003, *n* = 40) (Table 3).

**Table 3:** Morphometric data of the left and right calcanei

	*N*	Left		Right	
Metric assessment (cadaveric specimen)					
PF length (in mm)^a^	40	31.7	(3.5)	32.2	(3.6)
PF width (in mm)^a^	40	22.8*	(2.6)	23.5*	(2.8)
MF length (in mm)^b^	23	17.6	(2.3)	18.1	(2.5)
MF width (in mm)^b^	23	11.9	(1.4)	11.7	(1.5)
MF + AF length (in mm)^c^	10	32.0	(3.0)	31.7	(2.7)
MF + AF width (in mm)^c^	10	11.9	(1.1)	11.9	(1.6)
AF length (in mm)^b^	23	11.6	(1.9)	12.1	(2.4)
AF width (in mm)^b^	23	9.1	(1.4)	8.9	(1.5)
Distance PF–MF (in mm)^a^	40	6.6	(1.5)	6.6	(1.5)
Distance MF–AF (in mm)^b^	23	6.6	(2.1)	6.1	(2.2)
Intersection angle (in degrees)^d^	36	140.3	(8.4)	141.0	(8.5)
Cylinder fit parameters (CT data living individuals)					
Deviation angle (in degrees)^e^	20	−70.1	(5.6)	−68.6	(4.4)
Inclination angle (in degrees)^e^	20	−2.4	(6.9)	−4.1	(7.9)
Diameter (in mm)^e^	20	41.9	(7.4)	42.2	(8.2)

Numbers presented are averages (SD).

Abbreviations: AF, anterior facet; MF, middle facet; PF, posterior facet.

a Measured in Type A, B, and C.

b Measured in Type A.

c Measured in Types B and C.

d Measured after maceration of the bone.

e Adapted from Kleipool et al. (2019).

*P = 0.003: significant difference between left and right sides.

A significant difference was found in the intersection angle of the cadaveric specimens between the three calcanei types (ANOVA, *F* = 6.83, *P* = 0.002). Bonferroni post hoc analysis showed that intersection angle associated with Type A was significantly smaller (138.8 ± 8.5°) compared to Type C (147.2 ± 6.7°) (*P* < 0.001). A smaller angle corresponds to a steeper slope between the anterior and middle parts.

### Types of Coupled Tali and Calcanei

A large number of coupled cadaveric tali and calcanei, that is, the talus and calcaneus of one side of one individual, had noncorresponding types (Table 4). The frequency of noncorresponding types between the coupled tali and calcanei was as high as 48.8%. Of these noncorresponding combinations within the coupled bones, the Type A calcaneus was most frequently coupled with a Type B talus (36.3%).

**Table 4:** Cross tabulation of the combinations of types observed in coupled tali and calcanei (the talus and calcaneus of one side of one individual)

Calcaneus		Talus						Total calcaneus	
		Type A		Type B		Type C			
	Type A	**28.8%**	**(23)**	37.5%	(30)	–	–	*66.3%*	*(53)*
	Type B	1.3%	(1)	**13.8%**	**(11)**	–	–	*15.0%*	*(12)*
	Type C	–	–	6.3%	(5)	**12.5%**	**(10)**	*18.8%*	*(15)*
Total Talus		*30.0%*	*(24)*	*57.5%*	*(46)*	*12.5%*	*(10)*	*100%*	*(80)*

The diagonal with bold numbers represent the corresponding classifications between the calcaneus and talus. Off diagonal numbers represent the noncorresponding classifications. Numbers presented are the percentages of the total, and the numbers in parentheses represent the absolute number of specimen.

These results showed that many combinations are possible between the type of coupled tali and calcanei. The most frequently encountered combinations included calcaneus Type A and talus Type A, and the combination of calcaneus Type A with talus Type B.

### Osteoarthritic Changes

OA changes in the facets were found in 42.5% (*n* = 34) of the 80 cadaveric calcanei, and in 61.3% (*n* = 49) of the 80 cadaveric tali in the middle or anterior facet (Table 5). No OA changes were found on the posterior facet. The anterior part, on both the talus and calcaneus, was most frequently affected.

**Table 5:** Osteoarthritic changes found in the 80 calcanei and tali, specified per location, and per type for both calcanei and tali

	Calcaneus				Talus			
	MF		AF		MF		AF	
A	1.9%	(1)	43.4%	(23)	–	–	66.7%	(16)
B	16.7%	(2)	58.3%	(7)	2.2%	(1)	54.4%	(25)
C	–	–	20.0%	(3)	–	–	80.0%	(8)
Total	3.8%	(3)	41.3%	(33)	1.3%	(1)	61.3%	(49)

Numbers presented are the percentages of the total, and the numbers in parentheses represent the absolute number of specimen. Number of calcaneus: Type A = 53; Type B = 12; Type C = 15. Number of talus: Type A = 24, Type B = 46, and Type C = 10.

Abbreviations: AF, anterior facet; MF, middle facet.

GEE only demonstrated a significant association between the presence of OA changes and age for the calcaneus (OR = 1.38 per 5 years, *P* = 0.019, 95% confidence interval 1.1–3.2). No significant associations were demonstrated between the intersection angle, type of calcaneus, length or width of each facet, or corresponding types of coupled tali and calcanei and OA changes.

### Interobserver Analyses

#### *Types of bone*

Both observers individually classified the cadaveric calcanei and tali as Types A, B, or C based on the calcaneal and talar facets pattern of connection and number (Table 6, Fig. 1). There was complete agreement between both observers in the classification of Type A calcanei. Between the other types of calcanei (Types B and C) and all types of tali (Types A–C), there were also noncorresponding classifications. These noncorresponding classifications of the patterns of the calcaneus were of Types B and C (5%, *n* = 4). For the 19 tali, these were of Types A and B (18.8%, *n* = 15), and of Types B and C (5%, *n* = 4), thus a total of 23.8% (*n* = 19).

**Table 6:** Cross tabulation of the type (A, B, or C) of the cadaveric calcaneus and talus based on the patterns of calcaneal or talar facets classified by both observers

Observer 1		Observer 2						Total Observer 1	
		Type A		Type B		Type C			
Calcaneus	Type A	**66.3%**	**(53)**	–	–	–	–	*66.3%*	*(53)*
	Type B	–	–	**15.0%**	**(12)**	–	–	*15.0%*	*(12)*
	Type C	–	–	5.0%	(4)	**13.8%**	**(11)**	*18.8%*	*(15)*
Total Observer 2		*66.3%*	*(53)*	*20.0%*	*(16)*	*13.8%*	*(11)*	*100%*	*(80)*
Talus	Type A	**13.8%**	**(11)**	16.3%	(13)	–	–	*66.3%*	*(24)*
	Type B	2.5%	(2)	**53.3%**	**(41)**	3.8%	(3)	*15.0%*	*(46)*
	Type C	–	–	1.3%	(1)	**11.3%**	**(9)**	*18.8%*	*(10)*
Total Observer 2		*66.3%*	*(13)*	*68.8%*	*(55)*	*15.0%*	*(12)*	*100%*	*(80)*

The diagonal with bold numbers represent the corresponding classifications between the two observers. Off diagonal numbers represent the noncorresponding classifications. Numbers presented are the percentages of the total, and the numbers in parentheses represent the absolute number of specimen.

As there were discrepancies between the observed patterns between observers, which make it difficult to present the results, the classification of Observer 1 (Table 6, most right column) was used for analyses and reports as presented earlier.

#### *Metric data calcaneal facets*

The metric assessment of the cadaveric calcaneal facets included the width and length of the posterior, middle, and anterior facets, and the shortest distances between the facets. The ICCs between the two observers for these measurements of the calcaneus ranged from substantial to excellent (ICC 0.792–0.986) (Terwee et al., 2007) (Table 7).

**Table 7:** ICCs and the 95% confidence interval between the two observers for measurements on the cadaveric calcaneal facets

Measurement		ICC	95% Confidence interval
Length PF	(*n* = 80)	0.965	(0.940–0.978)
Width PF	(*n* = 80)	0.958	(0.932–0.974)
Length MF	(*n* = 80)	0.986	(0.979–0.991)
Width MF	(*n* = 80)	0.885	(0.820–0.926)
Length AF	(*n* = 53)	0.910	(0.833–0.950)
Width AF	(*n* = 53)	0.792	(0.642–0.880)
Minimal distance PF–MF	(*n* = 80)	0.867	(0.792–0.914)
Minimal distance MF–PF	(*n* = 53)	0.940	(0.896–0.965)

Abbreviations: AF, anterior facet; MF, middle facet; PF, posterior facet.

For the analysis of the morphometric data, the mean distance of both measurements of the two observers was used.

## DISCUSSION

This study assessed the bilateral symmetry of the subtalar articular joint facets and sought to find a (significant) relationship between the morphology and OA changes.

### Bilateral Symmetry

Overall, the results demonstrated that the articular facets in pattern, length, and width, and for the posterior facet also in orientation and curvature, are highly symmetrical. However, about one‐fifth of the individuals had a left–right asymmetry in the type of bone and a significant difference was found for the width of the posterior facet between left and right. Unfortunately, we do not have information about the laterality preference of the subjects to correlate these results. In a previous study, with a very high number of archeological calcanei, a frequency of left–right asymmetry in type of 10% was reported (Ragab et al., 2003). No literature is available for the symmetry of the type of talus.

The high degree of symmetry is in line with earlier studies that have demonstrated a high degree of symmetry in the lower extremity (Latimer and Lowrance, 1965). Specifically for the hindfoot, a strong degree of symmetry was demonstrated for the whole talus (Islam et al., 2014), the calcaneus (Stephan et al., 2014) and tibia, fibula, talus, and calcaneus (Tümer et al., 2018). A clear cause for the asymmetry in type of the left and right tali or calcanei presented here and in Ragab et al. (2003) is not at hand for now. Bunning and Barnett (1965) for the calcaneus and Rehman (2014) for the talus concluded that the patterns are probably genetically determined and are not developmental responses to walking habits, physique of a person, or duration of weight bearing in postnatal life. Rehman's (2014) results show that in the late fetal period already left–right differences are present in tali. The asymmetry could be a variation of the symmetrical nature of the embryologic body plan (Deng et al., 2015) and thus genetically driven, but possibly small environmental in utero differences, such as oligohydramnios or breech presentation, earlier in development might have caused the asymmetry. Be that as it may, this occurrence of asymmetry has to be taken into account by surgeons that use the contralateral noninjured side as a reference.

### Osteoarthritic Changes and Morphology

In the second part of this study, several morphological parameters were investigated to assess a possible relationship with the presence of OA changes. The congruency and stability of the joint are important determining factors in developing OA (Henak et al., 2013). The first parameter that was investigated was the type of calcaneus as Bruckner (1987) hypothesized that a three‐facet configuration (Type A) is more stable than a two‐facet configuration (Type B or C). This was confirmed by two subsequent studies that reported a significant higher frequency of OA changes in specimen with two facets (Type B or C) compared to specimens with three facets (Type A) (Drayer‐Verhagen, 1993; Madhavi et al., 2008). However, the reported frequencies of specimens with OA changes between these two studies and the present study differ greatly (Table 8).

**Table 8:** Frequencies of osteoarthritic changes as observed per type of calcaneus in two other studies and in the present study

	Three facets (Type A)		Two facets (Types B and C)	
Drayer‐Verhagen (1993)	35.3%	(*n* = 18/51)	65.4%	(*n* = 68/104)
Madhavi et al. (2008)	9.3%	(*n* = 4/43)	24.4%	(*n* = 39/160)
Present study	45.3%	(*n* = 24/53)	37.0%	(*n* = 10/27)

Even though the two other studies had incomparable frequencies, they still had the same conclusion. The present study could not demonstrate this relationship. A difference in the methodology by which OA changes were scored may, in part, explain the different reported frequencies between these three studies. The present study assessed the joints of wet preserved specimens with intact cartilage, whereas the other two studies used macerated and archeological bones without cartilage. The presence of OA in these latter two studies was thus based on secondary features associated with OA. In the present study, the cartilage itself was examined, thus enabling the identification of changes associated with the early onset of OA, a phase during which the associated changes examined in the previous studies has not yet developed.

A second parameter was the intersection angle which has also been linked to the stability of the subtalar joint and thus to OA changes (Drayer‐Verhagen, 1993; Madhavi et al., 2008). A smaller angle means a steeper slope between the middle and anterior part and potentially contributes to a higher stability. The present study found a significant difference between the intersection angle in Types A and C calcanei. The previous reports on the intersection angle by Drayer‐Verhagen (1993) and Madhavi et al. (2008) did not discriminate between Types B and C and pooled these types into one category (two‐facet configuration). Their averages differed significantly between Type A and B/C. In the present study, Type B was not significantly different from the other two types and the average angle was in between Types A and C. This can be addressed in future studies with a higher number of specimens. But also for this parameter, no significant interaction could be demonstrated.

A third parameter that was tested for an association with OA changes was whether the types corresponded in one coupled talus and calcaneus. No previous studies reported on the corresponding type of coupled calcaneal and talar facets in a European population. Corresponding or noncorresponding types could indicate a more or less congruent joint, respectively. Noncongruent joints are prone to develop OA (Henak et al., 2013). No significant association could be demonstrated. The most frequently affected part of the calcaneus and talus was the anterior part. Interestingly, no OA changes were observed in the posterior facet, which is in agreement with Madhavi et al. (2008).

Of the other parameters (such as lengths and widths of the facets) that were evaluated in a possible relationship with OA changes only age showed to be a significant factor. This was expected since aging and OA changes are highly correlated (Loeser, 2013).

### Interobserver Analyses

The interobserver analyses showed discrepancies between the observed types. Both observers agreed on all Type A calcanei. Types B and C are on a gliding scale. Jung et al. (2015) introduced a method to quantify the constriction, or the “degree of separation” as they called it, between the anterior and middle facets in Type B or C. This elegant method could not be used in our study as it was problematic to accurately identify the borders of the facets on the photographs of the wet specimen with intact periarticular soft tissue. The edges needed to be manually detected. The interobserver analysis of the metric data showed a substantial to excellent correlation. No metric analyses were performed for the tali. The boundaries of the middle and/or anterior facets could not be measured accurately. This is also reflected in the high number of noncorresponding classifications between both observers for the types of the tali. Considering our relative high frequency of discrepancies, we advise to include this analysis in future studies and to take into account some deviation from the presented distributions in current literature that did not present an interobserver analysis.

### Strengths, Limitations, and Recommendations

The strength of the present study was that we assessed paired hindfeet with intact cartilage, whereas most other studies used macerated or archeological specimens. We thereby could examine the cartilage itself and not only the subchondral surface when assessing for OA changes. It also allowed to look at the types within a coupled talus and calcaneus. Furthermore, we had a CT data set of healthy subjects for analyses. A limitation was the low number of cadaveric specimen and their high age. Another limitation was the measurement by sliding calipers of the facets. The posterior facet has a pronounced curvature. To deal with this, we used data from a previous study that did account for this 3D morphology. This limitation was also the case for the fused middle and anterior facets that in some specimen had a concave curvature. A similar approach with a geometrical shape fit as with the cylinder for the posterior facet might be applied to these facets, based on 3D laser scans or CT scanning. Furthermore, demographically differences that exist in the distribution of the types (Forriol Campos and Gomez Pellico, 1989) might also be present in the relationship between OA changes and morphology or left–right symmetry. Future studies can address these issues.

## CONCLUSIONS

Orthopedic and trauma surgeons can use this information to plan an operation using the contralateral noninjured foot in, for example, anatomical reduction or the size and shape of an implant. Continued research will potentially demonstrate an unambiguous relationship between the morphology and the stability or the risks for developing OA, since previous studies did find a significant relationship, but the present study did not (Drayer‐Verhagen, 1993; Madhavi et al., 2008).

In conclusion, the present study demonstrated a strong degree of bilateral symmetry in the morphology of the talocalcaneal joint facets. OA changes are frequently found, primarily at the anterior aspect of both calcanei and tali. However, only age had a significant interaction with the presence of OA changes. Other characteristics need further exploration.
